# Cardiac venous malformation concurrent with multiple hepatic venous malformations: A case report

**DOI:** 10.3389/fcvm.2022.1001996

**Published:** 2022-10-31

**Authors:** Shijie Zhang, Zhenqiang Xu, Chengwei Zou, Gang Zhang

**Affiliations:** ^1^Department of Cardiovascular Surgery, Shandong Provincial Hospital, Shandong University, Jinan, China; ^2^Department of Cardiovascular Surgery, Shandong Provincial Hospital, Shandong First Medical University, Jinan, China

**Keywords:** cardiac, cardiac venous malformation, hepatic venous malformation, cardiac metastases, tricuspid regurgitation

## Abstract

A 50-year-old woman who had previously undergone right radical mastectomy presented with chest tightness and shortness of breath after physical activities. A cardiac mass and multiple hepatic lesions were successively detected. We first performed hepatic puncture biopsy. Histopathologic examination confirmed that the multiple hepatic lesions were venous malformations. Based on the imaging findings and previous reports in the literature, we boldly speculated that the cardiac mass was also a venous malformation. The cardiac venous malformation was successfully resected, and the postoperative pathology confirmed our suspicion.

## Introduction

Cardiac cavernous hemangiomas, which should be now classified as venous malformations (VMs) (1), account for 5 to 10% of benign mass-forming cardiac lesions/tumors (2). Most cardiac VMs are solitary and concurrence at multiple locations is rarely reported (3). A literature review reveals cardiac VM coexisting VMs at other locations in the liver, lung, pleura, and thymus (4). Here, we report a rare right ventricular VM concurrent with multiple hepatic VMs. To our knowledge, only several cases of cardiac VMs have been reported coexisting with VMs in the liver (4–6). This case describes how we first identify the extracardiac VMs and then associate it with the intracardiac VM in a patient with breast cancer history. The diagnostic insight and experience are valuable for physician communication and learning.

## Case presentation

A 50-year-old female patient was admitted with chest tightness and shortness of breath after activities. The patient denied previous cardiovascular antecedents and family history. She reported a history of right radical mastectomy performed 10 years ago. She did not receive radiotherapy, chemotherapy, or endocrine treatment after the operation. On admission, the patient’s body temperature was normal (36.5°C) with a blood pressure of 139/70 mmHg, and a heart rate of 84 bpm. The patient was 158 cm tall and weighed 61.3 kg.

Physical examination revealed a grade III/VI systolic murmur at the left sternal margin. Jugular venous distention, generalized edema, and shifting dullness indicated severe right heart failure. The liver was enlarged, exceeding one transverse finger below the costal margin. By palpation, the liver edge felt slightly firm. There was no tenderness or rebound pain in the abdomen. The patient did not complain of other symptoms. The physical examination showed no positive signs of other systems.

Electrocardiography showed sinus rhythm, right axis deviation, and incomplete right bundle branch block (Supplementary Figure 1).

## Laboratory examination

Routine blood examination: Hemoglobin level was 147 g/L; hematocrit level was 43.3%; and serum leukocyte and platelet levels were 3.93 × 10^9^/L and 175 × 10^9^/L, respectively. The biochemical investigation showed no alteration in the following serum levels: liver enzymes, albumin, urea, creatinine, potassium, glucose, and sodium. Direct and indirect bilirubin levels were elevated (20.89 and 33.88 μmol/L, respectively). Myocardial enzymes and myocardial markers including creatine kinase (CK), creatine kinase isoenzyme mass (CK-MB), myoglobin (MYO), and high-sensitivity troponin T (HS-TnT) were normal. B-type natriuretic peptide (BNP) level was 3,732.00 pg/mL. The blood tests for tumor markers were not evident except for an elevated CA125 level of 240 U/mL.

## Imaging results

**FIGURE 1:**
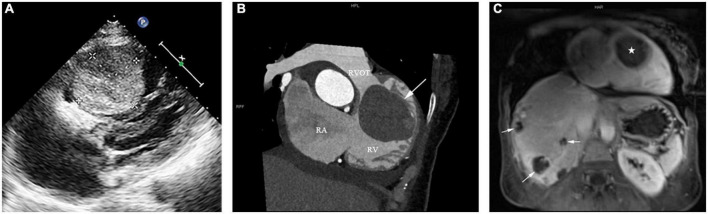
**(A)** Preoperative transthoracic echocardiography showing a large mass measuring 4.82 cm × 4.48 cm × 6.42 cm attached to the anterior wall of the right ventricle, with a moderate to strong and relatively homogeneous echo. **(B)** The computed tomography scan of the patient’s chest showing a right ventricular mass (arrow) in the right ventricular outflow tract (RVOT). RA: right atrium; RV: right ventricle. **(C)** Reconstructed magnetic resonance imaging of the patient’s chest and abdomen showed a cardiac mass concurrent with multiple hepatic lesions. The right ventricular mass and the multiple liver lesions are indicated by a star and three arrows, respectively.

Echocardiography revealed a large solid mass (4.82 cm × 4.48 cm × 6.42 cm) (Figure 1A), with right ventricular outflow tract obstruction and secondary severe tricuspid regurgitation (Video 1). No abnormal echoes were detected in the pericardium or pericardial cavity. Computed tomography (CT) scan revealed right ventricular mass (Figure 1B), along with multiple occupancies in the liver. A diagnosis of breast cancer metastasis was suspected at first. Magnetic resonance imaging (MRI) was performed to further define the mass of these two organs. The cardiac mass (Figure 1C) showed iso-intensity on T1-weighted images and hyper-intensity on T2-weighted images compared with the normal myocardium of the right ventricle. The mass showed diffuse contrast uptake with gadolinium enhancement. Further, evaluation of the liver revealed multiple mass lesions with varied sizes showing progressive enhancement, indicating a typical VM (Figure 1C). Notably, the lesions of two organs were not detected in previous assessments of breast cancer.

Brain MRI and abdominal ultrasound revealed no other lesions. Coronary angiography (CA) revealed no obvious stenosis in the coronary arteries and no discernible tumor-feeding artery was detected.

## Management

**FIGURE 2:**
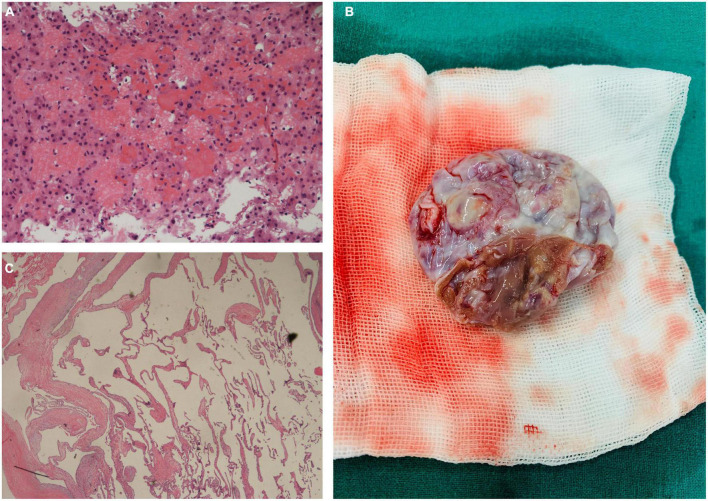
**(A)** Pathological examination of the liver mass showing multiple irregular dilatated vascular spaces and layers of endothelial cells without signs of malignancy (Hematoxylin and eosin, × 200). **(B)** Intraoperative photograph of the excised specimen appeared as a cystic mass with a broad base and intact capsule. **(C)** Pathological evaluation of the cardiac mass (Hematoxylin and eosin, × 100).

Although homologous multiorgan lesions were suspected, they were not determined as benign or malignant. The patient underwent hepatic puncture biopsy under ultrasound localization. Results of the pathological examinations revealed hepatic VM (Figure 2A). Given her medical history and imaging findings, the diagnosis of cardiac VM and multiple VMs was highly suspected. We decided to perform a resection of the cardiac mass.

Before surgery, our team administered diuresis, heart-rate reduction, and nutritional myocardial therapy. The patient’s symptoms and cardiac function improved significantly. Complete excision of the cardiac mass (Figure 2B) was performed under standard cardiopulmonary bypass. The pedicle of the mass was attached to the interventricular septum, near the anterior wall of the right ventricle. The capsule was intact, and there was no adhesion or fusion with the tricuspid valve and chordae tendinae. We completely excised the pedicle from the myocardium while preserving the ventricular structure, as well as the free wall of the right ventricle. Upon exploration, the valve annulus of the tricuspid valve was enlarged. We believe that the cardiac lesion blocked the right ventricular outflow tract, causing dilatation of the right ventricle and enlargement of the valve annulus, resulting in functional tricuspid insufficiency. We used the 28 # Edwards tricuspid annuloplasty ring to perform tricuspid valvuloplasty. Pathological examination demonstrated thin-walled interanastomosing channels lined by endothelial cells. There is no atypia, necrosis or active mitosis (Figure 2C). By immunohistochemistry, the lining cells are positive for CD31 and CD34, confirming endothelial differentiation. Based on the histological and immunohistochemical features, we diagnosed the cardiac mass as a VM. The patient was discharged 6 days after the operation.

Although multiple VMs in the liver were also detected, they were small in size. Nonetheless, the patient complained of no abdominal symptoms. The liver enlargement disappeared, and her liver function was normal. We recommended a regular follow-up. A telephone follow-up was conducted 6 months and 1 year after surgery. The patient had no discomfort and her tolerance to activity was good. In addition, cardiac echocardiography revealed no tumor recurrence or tricuspid regurgitation. No significant progression of the hepatic VMs was detected.

## Discussion

Diagnosis of the cardiac VM is aided by imaging techniques. Echocardiography has become an important tool for the initial identification of cardiac VMs. Contrast-enhanced myocardial echocardiography has evolved rapidly in recent years and utilized in the diagnosis of cardiac vascular malformation (7). CT and MRI can be used to evaluate the size, location, extracardiac extension, and the degree of myocardial involvement of the malformation. In addition, MRI in this study suggested that the lesions were of vascular origin, which facilitated the diagnosis and differential diagnosis. Typically, cardiac VM shows isointense and high signal intensity compared with myocardium in T1- and T2-weighted images, respectively (5, 8, 9). Homogeneous enhancement after contrast infusion is another typical manifestation of cardiac VM (8–10). The high vascularity of cardiac VM is of great diagnostic value clinically. Besides, CA can be used to evaluate the presence of feeding vessels. However, imaging examination only provides limited diagnostic clues for cardiac VM. Biopsy is needed for a definitive diagnosis.

In this case, the patient was first diagnosed with a right ventricular mass, and further examination revealed concurrent multiple liver masses. Given that right ventricular occupying lesions are generally malignant, and both cardiac (11) and hepatic (12) metastases of breast cancer have been reported, it was initially believed that the cardiac mass, in this case, was malignant. However, as the diagnosis progressed, we assumed that the cardiac lesion was more likely to be benign. First, the mass was confined to the right ventricle, without obvious myocardial infiltration or invasion of other cardiac chambers, pericardium, or pericardial cavity. Both imaging and pathological findings showed that multiple liver lesions were VMs. The possibility of multiple metastases from breast cancer was ruled out. Finally, as mentioned above, cases of cardiac VM concurrent with hepatic VM have been reported in the literature. Despite the patient’s history of breast cancer as a confounding factor, preoperatively, we believed that the cardiac mass was likely consistent with the nature of the lesions in the liver, which was also a VM. Total resection is possible in most cardiac VMs, but a few cases cannot be excised (10). The diagnosis of previously reported cases of cardiac VMs was almost established by biopsy of surgically excised specimens, suggesting that the benign or malignant nature of the lesion was uncertain before surgery. Our team first identified the multiple liver mass as VMs pathologically Combined with the imaging findings, the mass in the heart was suspected as a VM. The postoperative pathology confirmed our suspicion.

The cause of VMs remains unclear. Previous studies have shown that somatic mutations in TIE2, PIK3CA, MAP3K3 and other genes lead to VMs (13–17). Although abnormal veins are present at birth, deep lesions often grow within the body and remain undetected until symptoms appear (18). VMs can progress rapidly in response to changes in hormone levels (during puberty or pregnancy), infection, trauma, and inappropriate treatment (19, 20). However, the etiology of cardiac VMs is rarely reported. In our case, the lesions of two organs were not detected in previous assessments of breast cancer. The patient did not receive chemotherapy or endocrine therapy after surgery for breast cancer. We speculate that the progression of VMs may be related to her perimenopausal period.

In summary, we reported a rare case of right ventricular VM concurrent with multiple hepatic VMs. The decision to operate the patient was based on relatively clear diagnosis of the nature of the lesion. In addition to complete resection of the right ventricular VM, we also performed a successful repair of the tricuspid valve. Long-term follow-up is required, especially to evaluate recurrence and right heart function.

Despite its rarity, clinicians need to be aware that cardiac VMs are occasionally associated with extracardiac VMs. However, any underlying mechanism is unknown.

## Patient perspective

“As a patient, I am glad that the cause of the disease has been found and solved. I underwent radical surgery for breast cancer 10 years ago. In recent years, I haven’t seen my breast surgeon again. At first, I thought the breast cancer had returned and metastasized, which frightened me. Fortunately, both the cardiac and hepatic tumors were benign. I am very grateful to the doctors and nurses who treated me.”

## Data availability statement

The original contributions presented in the study are included in the article/Supplementary material, further inquiries can be directed to the corresponding author/s.

## Ethics statement

Written informed consent was obtained from the individual(s) for the publication of any potentially identifiable images or data included in this article. Written informed consent was obtained from the patient for the publication of this case report.

## Author contributions

SZ and ZX cared for the patient and wrote the final manuscript. CZ and GZ revised it critically. All authors read and approved the final version of the manuscript.

## Supplementary material

Supplementary Figure 1 The electrocardiography result.
