# Supplementary figures and images for: Cardiac venous malformation concurrent with multiple hepatic venous malformations: A case report
Source: Front Cardiovasc Med. 2022 Oct 31;9:1001996. doi: 10.3389/fcvm.2022.1001996 (PMC9659562; doi:10.3389/fcvm.2022.1001996)

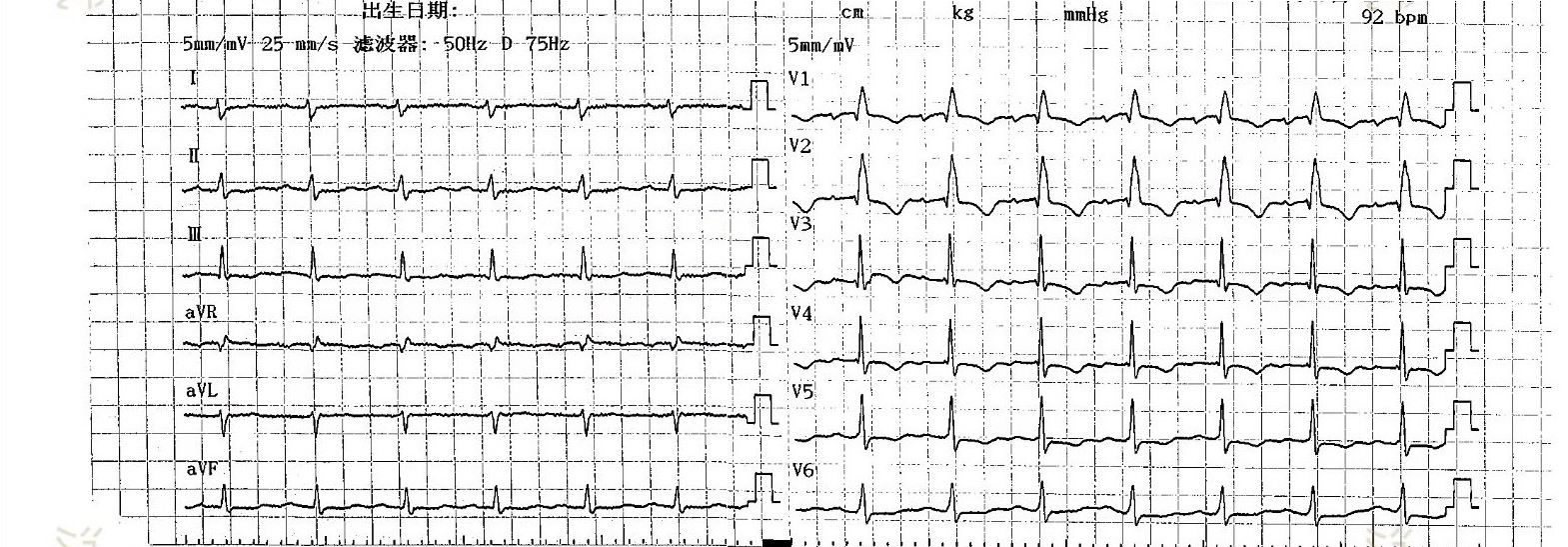

Supplement: Supplementary Figure 1 — The electrocardiography result. [file Image_1.TIF]
